# Clinical Profile and Short-Term Outcomes of Acute Kidney Injury in Elderly Patients in a Tertiary Care Center

**DOI:** 10.7759/cureus.62690

**Published:** 2024-06-19

**Authors:** Govind Prasad, Praphull Deepankar, Manoj Kumar Choudhary, Arshad Ahmad, Bhim Ram, Naresh Kumar, Prem S Patel

**Affiliations:** 1 Department of General Medicine, Indira Gandhi Institute of Medical Sciences, Patna, IND; 2 Department of Nephrology, Indira Gandhi Institute of Medical Sciences, Patna, IND

**Keywords:** tertiary care center, mortality, short-term outcomes, clinical profile, elderly, acute kidney injury

## Abstract

Background

Acute kidney injury (AKI) is a common and serious condition affecting elderly patients. Despite its significance, comprehensive research focusing specifically on the clinical profile and short-term outcomes of AKI in this vulnerable population is lacking.

Objective

This study aimed to evaluate the clinical profile and short-term outcomes of AKI in elderly patients admitted to a tertiary care center.

Methods

A prospective observational study was conducted from December 2023 to March 2024, involving 75 elderly patients (aged ≥65 years) diagnosed with AKI. Baseline demographic data, clinical profiles, laboratory investigations, mortality rate among elderly patients diagnosed with AKI within 30 days of diagnosis, and short-term outcomes were recorded and analyzed. Descriptive statistics and appropriate statistical tests were used for the data analysis.

Results

The study cohort had a mean age of 72.6 years. Hypertension was present in 55 patients (73.3%), and diabetes mellitus was observed in 30 patients (40.0%). Prerenal causes of AKI were identified in 40 patients (53.3%), while acute tubular necrosis was found in 25 patients (33.3%). Stage 2 AKI was the most common, affecting 35 patients (46.7%). Out of the 75 patients, 15 patients (20.0%) succumbed to AKI within the study period. Deceased patients had longer hospital stays, with a median of 16 days compared to 10 days for survivors. ICU admission was required for 13 of the deceased patients (86.7%), compared to 32 of the surviving patients (53.3%). The need for renal replacement therapy was higher among the deceased patients, with 11 out of 15 patients (73.3%) requiring it, compared to 19 out of 60 surviving patients (31.7%). Renal function recovery was notably lower in the deceased patients.

Conclusion

AKI in elderly patients was associated with significant morbidity and mortality, highlighting the need for early recognition, appropriate management, and preventive strategies. A comprehensive evaluation of the clinical profile and short-term outcomes of AKI in the elderly population provides valuable insights for optimizing patient care and improving outcomes.

## Introduction

Acute kidney injury (AKI) is a significant and potentially life-threatening condition characterized by a sudden decline in renal function, resulting in the accumulation of metabolic waste products and electrolyte imbalances [1]. Elderly individuals are particularly vulnerable to AKI owing to age-related physiological changes, comorbidities, and polypharmacy, which predispose them to kidney injury and adverse outcomes [2]. Despite advancements in medical care, the incidence of AKI in the elderly continues to rise, posing substantial clinical and economic burdens on healthcare systems worldwide [3].

The elderly population, defined as individuals aged 65 years and above, constitutes a rapidly growing demographic segment globally [4]. With aging, there is a natural decline in renal function, manifested by alterations in renal blood flow, glomerular filtration rate, and tubular function [5]. These age-related changes render elderly individuals more susceptible to AKI triggers such as hypovolemia, nephrotoxic medications, and sepsis, further exacerbating the risk of kidney injury [6]. Additionally, the presence of multiple comorbidities, including hypertension, diabetes mellitus, and cardiovascular disease, contributes to the pathogenesis and progression of AKI in this population [7].

Despite the high prevalence and clinical significance of AKI in the elderly, there remains a paucity of literature specifically focusing on this vulnerable subgroup. Existing studies often include heterogeneous populations or focus on the specific etiologies of AKI, limiting their generalizability to elderly patients [8]. Therefore, there is a compelling need for comprehensive research to elucidate the clinical profile, etiology, and outcomes of AKI, specifically in elderly individuals.

This study aimed to address this knowledge gap by conducting a detailed evaluation of the clinical profile and short-term outcomes of AKI in elderly patients admitted to a tertiary care center. By systematically analyzing baseline characteristics, etiology, severity, and clinical outcomes, this study sought to provide valuable insights into the epidemiology, pathophysiology, and management of AKI in the elderly population. The findings of this study have the potential to inform clinical practice guidelines, optimize patient care strategies, and ultimately improve outcomes for elderly individuals at risk of AKI.

## Materials and methods

This prospective observational study was conducted at the Indira Gandhi Institute of Medical Sciences, Patna, India, from December 2023 to March 2024 to evaluate the clinical profile and short-term outcomes of AKI in elderly patients. The study protocol was approved by the Institutional Review Board (IRB) before commencement, and informed consent was obtained from all participants or their legally authorized representatives.

The study protocol adhered to the principles outlined in the Declaration of Helsinki and was approved by the IRB or Ethics Committee of the tertiary care center. Informed consent was obtained from all participants or their legally authorized representatives before enrollment in the study. Patient confidentiality was strictly maintained throughout the study, and the data were anonymized during analysis and reporting. A meticulous approach was used to ensure the enrollment of eligible participants in the study. A comprehensive review of medical records and electronic databases was conducted to identify 75 elderly patients aged 65 years and above who were diagnosed with AKI during the stipulated study period from December 2023 to March 2024.

The sample size was calculated using the following formula: n = Z^2^⋅p⋅(1−p)/d^2^, where n = required sample size; Z = Z-value (Z-score) corresponding to the desired confidence level (e.g., 1.96 for 95% confidence); p = estimated proportion of the population (e.g., expected mortality rate); and d = margin of error (precision).

Consecutive enrollment was employed to minimize selection bias, whereby eligible patients who met the inclusion criteria were approached for participation in the study. Informed consent was obtained from all participants or their legally authorized representatives before enrollment.

In this study, inclusion criteria were defined to identify eligible participants. Patients aged 65 years and above were included to maintain homogeneity in the study population, focusing specifically on elderly individuals. Additionally, a diagnosis of AKI was required for inclusion, with diagnostic criteria based on established guidelines such as the Kidney Disease: Improving Global Outcomes (KDIGO) criteria. These guidelines consider various factors, including changes in serum creatinine levels, urine output, and the duration of kidney injury.

Conversely, exclusion criteria were implemented to ensure the study’s focus on AKI in elderly patients and to minimize confounding factors. Patients with ESRD/CKD stage V patients on dialysis treatment were excluded, as were individuals with preexisting end-stage renal disease (ESRD) on chronic dialysis. This exclusion aimed to maintain homogeneity in the study population and to avoid the influence of distinct clinical characteristics and management considerations associated with advanced CKD and ESRD.

Furthermore, patients with incomplete medical records were also excluded from the study. This criterion was essential to maintain the integrity and reliability of the study findings by ensuring that all necessary data for the study objectives were available. By applying these inclusion and exclusion criteria, the study aimed to create a well-defined cohort of elderly patients with AKI, facilitating a comprehensive analysis of their clinical profile and short-term outcomes.

The screening process involved a thorough review of the patient’s medical records, including admission notes, laboratory results, and physician orders, to identify potential candidates that met the inclusion criteria. The identified patients were approached by the research team, and the study objectives, procedures, and potential risks and benefits were explained in detail. Informed consent was obtained from eligible patients or their legally authorized representatives before enrollment in the study. Baseline demographic information was meticulously collected for each participant to establish a comprehensive understanding of the study population. The following details were recorded: the age of each participant was documented to assess the distribution and characteristics of the elderly population, and sex distribution was noted to evaluate potential differences in AKI presentation and outcomes between male and female participants. Comorbidity information regarding preexisting medical conditions, such as hypertension, diabetes mellitus, and cardiovascular disease, was obtained to assess their impact on AKI development and prognosis.

Prerenal AKI was defined based on a clinical assessment and laboratory findings indicative of decreased renal perfusion. Specifically, prerenal AKI was identified by a fractional excretion of sodium (FeNa) <1%, a low urinary sodium concentration (<20 mmol/L), and a positive response to fluid resuscitation. Intrinsic AKI (acute tubular necrosis, or ATN) was defined as ATN based on urinary sediment analysis showing granular casts and renal tubular epithelial cells. Additionally, FeNa >2% and a lack of response to fluid resuscitation further supported the diagnosis of intrinsic AKI. Postrenal AKI was determined using imaging techniques, including ultrasound and CT scans, to identify any obstruction in the urinary tract, such as hydronephrosis or other obstructive uropathies. These definitions and diagnostic criteria were consistently applied across all patients to ensure an accurate classification of the etiology of AKI.

Detailed clinical profiles were compiled for the study cohort. Pathology diagnosis was not employed; instead, clinical observations, expert judgment, and specific laboratory tests were utilized to determine prerenal, intrinsic, and postrenal factors contributing to renal failure. The severity of AKI was assessed and classified according to the KDIGO staging criteria, which considers changes in serum creatinine levels, urine output, and duration of kidney injury. The presence or absence of sepsis, a common complication associated with AKI, was ascertained to evaluate its effect on AKI outcomes and management strategies. The requirement for renal replacement therapy, such as hemodialysis or continuous renal replacement therapy, has been documented to assess the severity and management of AKI in elderly patients.

Comprehensive laboratory investigations were performed on admission to characterize the biochemical and physiological parameters associated with AKI. The following laboratory parameters were measured: serum creatinine levels were measured to assess renal function and to determine the severity of kidney injury. Blood urea nitrogen levels were measured, as was the degree of uremia. Serum electrolyte levels, including sodium, potassium, and chloride, were measured to identify electrolyte imbalances commonly associated with AKI. Urine output was monitored to evaluate renal function and assess the severity of the kidney injury. Short-term outcomes following AKI diagnosis were meticulously recorded to assess the clinical course and prognosis of the elderly patients. The following outcomes were documented: the duration of hospitalization from AKI diagnosis to discharge was recorded to evaluate resource utilization and healthcare costs, and the need for ICU admission following AKI diagnosis was noted to assess the severity of illness and resource utilization. The occurrence of mortality within 30 days of AKI diagnosis was documented to evaluate short-term mortality rates and identify factors associated with a poor prognosis.

The primary outcome measure of this study was the mortality rate among elderly patients diagnosed with AKI within 30 days of diagnosis. Mortality was defined as death occurring within a specified time frame after an AKI diagnosis. The primary objective was to assess the short-term survival outcomes of elderly patients with AKI, providing valuable insights into disease severity and prognosis in this population. In addition to mortality, several secondary outcome measures were evaluated to comprehensively assess the clinical course and outcomes of AKI in elderly patients. The secondary outcome measures were as follows: the duration of hospitalization from the time of AKI diagnosis to discharge was recorded to assess the impact of AKI on healthcare resource utilization and the burden of illness on patients. The need for admission to the ICU following the diagnosis of AKI was documented to evaluate the severity of the illness and the intensity of medical care required for managing AKI-related complications. The requirement for renal replacement therapy, such as hemodialysis or continuous renal replacement therapy, was assessed to determine the severity of kidney injury and the extent of renal dysfunction necessitating advanced therapeutic interventions. The recovery of renal function at the time of discharge from the hospital was evaluated to determine the extent of renal recovery following the acute phase of AKI. Renal recovery was assessed based on improvements in serum creatinine level, urine output, and overall renal function.

Data were analyzed using IBM SPSS Statistics for Windows, Version 24.0 (Released 2016; IBM Corp., Armonk, NY, USA). Descriptive statistics were used to summarize the baseline characteristics and clinical outcomes. Continuous variables are expressed as mean ± standard deviation or median (interquartile range) based on the distribution, while categorical variables are presented as frequencies and percentages. Comparisons between groups were performed using appropriate statistical tests (t-test, chi-square test, and Mann-Whitney U test), with a significance level set at p < 0.05.

## Results

Table 1 illustrates the baseline characteristics of the elderly patients diagnosed with AKI in the study.

**Table 1:** Baseline characteristics of elderly patients with AKI AKI, acute kidney injury

Characteristic	Total (n = 75)	Deceased (n = 15)	Survivors (n = 60)	p-value
Age (years), mean ± SD	72.6 ± 6.8	75.4 ± 7.2	71.8 ± 6.5	0.037
Gender				
Male	40 (53.3%)	10 (66.7%)	30 (50.0%)	0.649
Female	35 (46.7%)	5 (33.3%)	30 (50.0%)	
Hypertension				
Yes	55 (73.3%)	14 (93.3%)	41 (68.3%)	0.012
No	20 (26.7%)	1 (6.7%)	19 (31.7%)	
Diabetes mellitus				
Yes	30 (40.0%)	12 (80.0%)	18 (30.0%)	0.021
No	45 (60.0%)	3 (20.0%)	42 (70.0%)	
Cardiovascular disease				
Yes	25 (33.3%)	9 (60.0%)	16 (26.7%)	0.137
No	50 (66.7%)	6 (40.0%)	44 (73.3%)	

The mean age of the total cohort was 72.6 years, with a slight variation between deceased patients (mean age: 75.4 years) and survivors (mean age: 71.8 years). Hypertension was prevalent among 55 (73.3%) of the patients overall, with a significantly higher proportion among deceased patients (14; 93.3%) than among survivors (41; 68.3%). Similarly, diabetes mellitus was present in 30 (40.0%) of patients, with a higher prevalence among deceased patients (12; 80.0%) than among survivors (18; 30.0%). There were no significant differences in sex distribution or the presence of cardiovascular disease between the deceased and survivor groups.

Table 2 outlines the etiology and severity of AKI in elderly patients.

**Table 2:** Etiology and severity of AKI AKI, acute kidney injury; ATN, acute tubular necrosis; KDIGO, Kidney Disease: Improving Global Outcomes

Etiology	Total (n = 75)	Deceased (n = 15)	Survivors (n = 60)	p-value
Prerenal				
Frequency	40 (53.3%)	8 (53.3%)	32 (53.3%)	0.081
Intrinsic (ATN)				
Frequency	25 (33.3%)	7 (46.7%)	18 (30.0%)	0.357
Postrenal				
Frequency	10 (13.3%)	0 (0.0%)	10 (16.7%)	0.029
Severity (KDIGO staging)				
Stage 1	15 (20.0%)	2 (13.3%)	13 (21.7%)	0.164
Stage 2	35 (46.7%)	8 (53.3%)	27 (45.0%)	0.046
Stage 3	25 (33.3%)	5 (33.3%)	20 (33.3%)	0.091

Most AKI cases were attributed to prerenal causes (40; 53.3%), followed by intrinsic causes, notably ATN (25; 33.3%). Postrenal causes were less common, accounting for 10 (13.3%) cases. Regarding the severity of AKI based on KDIGO staging, stage 2 AKI was the most prevalent (35; 46.7%), followed by stage 3 (25; 33.3%) and stage 1 (15; 20.0%). While there were no significant differences in etiology between the deceased and survivor groups, a higher proportion of deceased patients had stage 2 AKI than survivors, 8 (53.3%) vs. 27 (45.0%).

Table 3 presents the clinical outcomes of elderly patients diagnosed with AKI.

**Table 3:** Clinical outcomes of elderly patients with AKI AKI, acute kidney injury

Outcome measure	Total (n = 75)	Deceased (n = 15)	Survivors (n = 60)	p-value
Mortality (%)	20 (26.7%)	15 (100.0%)	-	<0.001
Length of hospital stay (days), median (IQR)				
Median (IQR)	12 (9-16)	16 (12-20)	10 (8-14)	0.003
Requirement for ICU admission (%)				
Yes	45 (60.0%)	13 (86.7%)	32 (53.3%)	0.015
No	30 (40.0%)	2 (13.3%)	28 (46.7%)	
Need for renal replacement therapy (%)				
Yes	30 (40.0%)	11 (73.3%)	19 (31.7%)	0.002
No	45 (60.0%)	4 (26.7%)	41 (68.3%)	
Recovery of renal function (%)				
Yes	60 (80.0%)	10 (66.7%)	50 (83.3%)	<0.001
No	15 (20.0%)	5 (33.3%)	10 (16.7%)	

The median length of hospital stay was significantly longer for the deceased patients (16 days) than for the survivors (10 days). Furthermore, a higher percentage of deceased patients required ICU admission (13; 86.7%) than survivors (32; 53.3%). Similarly, a greater proportion of deceased patients required renal replacement therapy (11; 73.3%) than survivors (19; 31.7%). Notably, renal function recovery was significantly lower among deceased patients (10; 66.7%) than among survivors (50; 83.3%).

**Figure 1:**
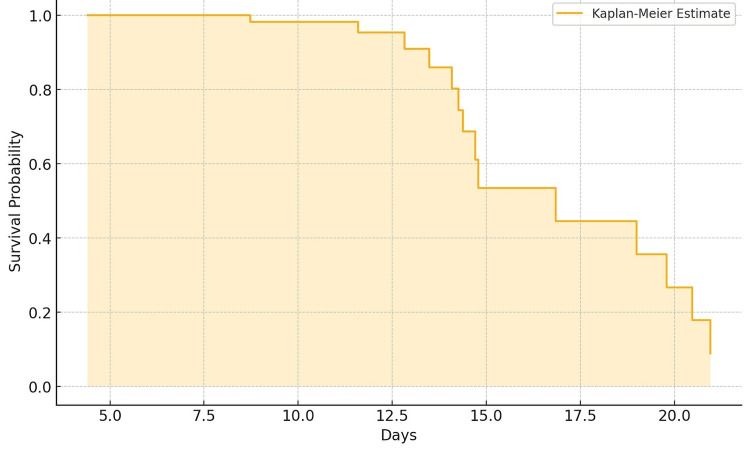
Kaplan-Meier curve for the data of elderly patients with AKI AKI, acute kidney injury

The Kaplan-Meier curve presented illustrates the survival probability over time for a simulated cohort of elderly patients diagnosed with AKI. The x-axis represents the number of days, while the y-axis indicates the survival probability. The step plot shows the estimated survival function, which decreases over time, reflecting the proportion of patients who survive beyond each time point. The shaded area around the curve represents the confidence intervals, providing a measure of uncertainty around the survival estimates. The curve shows a notable decline, particularly within the first 20 days, highlighting the critical period for patient survival post-AKI diagnosis (Figure 1).

## Discussion

AKI represents a significant clinical challenge, particularly in elderly patients, owing to its association with high morbidity and mortality rates [1]. This study aimed to comprehensively evaluate the clinical profile and short-term outcomes of AKI in elderly patients admitted to a tertiary care center. Through meticulous data collection and analysis, valuable insights were gained into the etiology, severity, and clinical course of AKI in this vulnerable population [9,10]. This discussion will delve into the key findings of the study, their implications for clinical practice, and areas for future research.

The etiology of AKI in elderly patients is multifactorial, often involving a combination of prerenal, intrinsic, and postrenal causes [11]. In this study, prerenal causes were the most common, reflecting the high prevalence of conditions such as volume depletion and hypoperfusion in elderly individuals. Notably, postrenal causes were absent among deceased patients, highlighting the importance of prompt recognition and management of obstructive uropathy to prevent adverse outcomes. Additionally, comorbidities such as hypertension and diabetes mellitus were identified as significant risk factors for AKI development, consistent with previous literature [12,13].

The severity of AKI, as assessed by the KDIGO staging criteria, plays a pivotal role in predicting clinical outcomes and mortality risk. Stage 2 AKI was more prevalent among the deceased patients, indicating a higher degree of kidney injury and associated complications. The association between AKI severity and mortality underscores the importance of early risk stratification and aggressive management strategies to mitigate adverse outcomes [14]. Moreover, advanced age was identified as an independent predictor of mortality, consistent with previous studies demonstrating an increased mortality risk in elderly patients with AKI [15,16].

The clinical outcomes of elderly patients with AKI were characterized by prolonged hospitalization, higher rates of ICU admission, and increased requirements for renal replacement therapy. Deceased patients had significantly longer lengths of hospital stay, reflecting the severity of the illness and the complexity of managing AKI-related complications. Furthermore, the need for ICU admission and renal replacement therapy was more prevalent among deceased individuals, highlighting the critical nature of their condition and the challenges associated with its management. A study by Chaïbi et al. found that severe AKI in critically ill patients was linked to a high mortality rate within the first two months. However, long-term follow-up showed a lower mortality rate. Among the long-term survivors, a quarter experienced worsening renal function and a significant decline in quality of life [17].

The findings of this study have several implications for clinical practice. First, early recognition and management of AKI in elderly patients are paramount to prevent disease progression and improve outcomes. Clinicians should maintain a high index of suspicion for AKI in elderly individuals, particularly those with underlying comorbidities such as hypertension and diabetes mellitus. Timely interventions, including fluid resuscitation, correction of electrolyte imbalances, and avoidance of nephrotoxic medications, are crucial for mitigating the risk of AKI development [18]. Second, risk stratification based on AKI severity and prognostic assessment is essential for guiding clinical decision-making and resource allocation. Patients with severe AKI, particularly those requiring ICU admission and renal replacement therapy, should receive prompt and aggressive management to optimize outcomes. Close monitoring of renal function and fluid status, along with early nephrology consultation, may help identify patients at high risk of adverse outcomes and facilitate timely intervention [19].

Finally, multidisciplinary collaboration and coordinated care are needed to address the complex needs of elderly patients with AKI. Integrated care pathways involving nephrologists, intensivists, geriatricians, and allied healthcare professionals can optimize patient management and enhance clinical outcomes. Emphasis should be placed on patient-centered care, including shared decision-making and advance care planning, to align treatment goals with patient preferences and values [20].

Despite its strengths, this study had several limitations that warrant consideration. First, the retrospective nature of the study may have introduced bias and limited the generalizability of the findings. Prospective multicenter studies with larger sample sizes are needed to validate the results and elucidate additional factors influencing AKI outcomes in elderly patients. Second, the study focused on short-term outcomes, and long-term follow-up data were not available. Future research should explore the impact of AKI on long-term morbidity, mortality, and renal function in the elderly population. Additionally, this study did not assess the effectiveness of specific interventions or treatment modalities in improving AKI outcomes. Randomized controlled trials evaluating novel therapeutic strategies and preventive measures are warranted to address this gap in knowledge and inform evidence-based practice.

Incorporating diagnostic methodologies such as urine electrolytes or urine microscopy to distinguish between prerenal and intrinsic causes could enhance the accuracy of AKI diagnosis in future studies. Elaborating on post-obstructive causes in the context of urinary retention is also crucial, as it could provide insights into the complexity of AKI etiology and guide tailored management strategies.

## Conclusions

This investigation illuminates senior AKI clinical characteristics and the short-term prognosis. These findings emphasize the necessity of early detection, risk stratification, and multidisciplinary therapy for AKI in this vulnerable population and its high morbidity and mortality. Clinicians should monitor older AKI patients and prioritize risk factor reduction and outcome optimization. Since elderly people often take multiple medications, the study could benefit from addressing and adjusting for potential confounding variables like provider care practices, patient socioeconomic status, and precise medication histories. Future research should address these limitations and improve our understanding of senior AKI pathogenesis, management, and long-term consequences.
